# Effect of Negative Ion Generation on Complex Plasma Structure Properties

**DOI:** 10.3390/molecules27248669

**Published:** 2022-12-08

**Authors:** Andrey V. Zobnin, Andrey M. Lipaev, Alexander D. Usachev

**Affiliations:** Joint Institute for High Temperatures, Russian Academy of Sciences, 125412 Moscow, Russia

**Keywords:** gas discharge, negative ions, complex plasma

## Abstract

We propose a low-density discharge plasma model that takes into account the impact of oxygen admixture in typical conditions of complex (dusty) plasmas. Numerical simulations based on this model show that the concentration of negative ions turns out to be very high, and they play an important role in the overall kinetics in this particular range of plasma conditions. The ambipolar diffusion electric field drags these negative ions into the center of the plasma. The density of negative ions is high enough to push the negatively charged dust component out of the center, both by weakening the radial electric field and by increasing the thermophoretic force. This phenomenon was observed in the published experiment and qualitatively supports the proposed model. Additionally, the proposed model allows an alternative explanation of the experiment.

## 1. Introduction

Complex plasmas are suspensions of micron or submicron solid particles in a “regular” plasma. These microparticles obtain charges due to difference of fluxes of electrons and ions on their surfaces. Charged particles strongly interact with each other and surrounding plasma and can exhibit such self-organization phenomena as crystallization [1] and formation of chains of microparticles (so-called string formation) [2,3].

**Figure 1:**
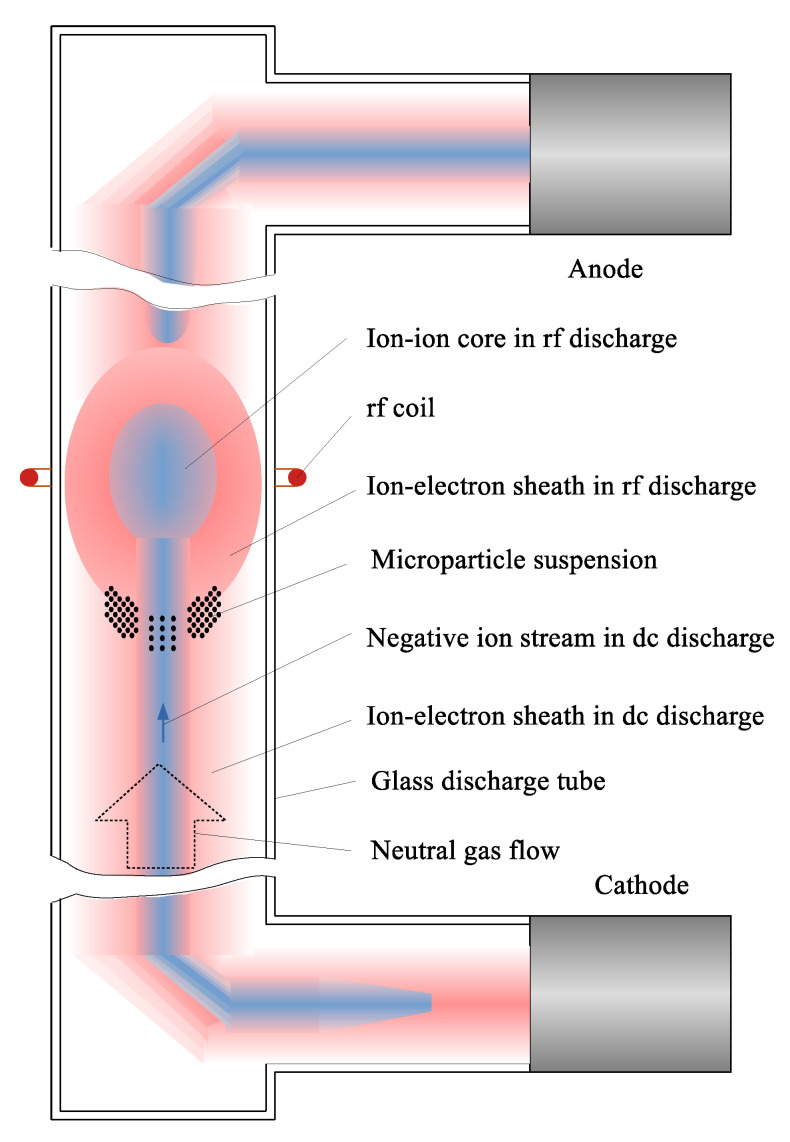
Sketch of the experimental set up used in [4].

The structures of the monodisperse 2.55 μm plastic spheres in a combined discharge, produced by coexisting DC and inductively coupled RF discharge (81.36 MHz, 1.6 W), were investigated in the paper [4]. The discharge was sustained in argon with 10% oxygen admixture at 30 Pa pressure in the ground-based discharge tube similar to the tube in the “Plasma Kristall–4” space facility [5]. The glass discharge tube had an inner diameter of 30 mm and a length of about 500 mm. The sketch of the experiment is shown in Figure 1. The gas mixture flow of 0.25 sccm prevented the system from plasma degradation. The discharge tube was vertically aligned in a way that gas flow partially compensates the microparticles weight. The de facto standard scanning laser sheet technique was used to obtain the 3D structure shape and microparticle distribution at different DC discharge currents, varied in irregular steps from 0 to 1.0 mA. Authors in [4] have demonstrated that the increase in the DC discharge current led to essential change in the structure of the microparticles cloud. In the pure RF discharge, the microparticle structure had a bowl-like shape and filled the low part of the RF discharge including the axial region more or less homogeneously. When a low DC discharge current was applied, the density of particles around the axis decreased distinctly. A further DC discharge current increase led to an even deeper decrease in the microparticle density in the central part of the dust cloud, up to the void formation. Additionally, the string-like structures of the microparticles became more and more pronounced as the discharge current increased.

The string formation cannot be attributed to increasing the positive ion flow velocity when the DC discharge was applied, because the electric field required for the microparticle levitation was determined by a balance of the electric and gravity forces and did not change essentially. The mean free path of ions under the experimental conditions was only about 0.14 mm, so the ion flow velocity was determined by a local electric field and could not change substantially during the experiment and cause the string formation.

In this paper, we propose an explanation of the experimental observations described in [4] by a compact axial stream of negative ions in a DC discharge doped with electronegative oxygen, as well as its influence on microparticle number density distribution and on a dust cloud structure.

## 2. Discharge Model

A typical low-power discharge used in complex plasma experiments features an electron density of $n_{e}≲10^{9}\;{\mathrm{cm}}^{-3}$ and a relatively long lifetime of negative ions. The negative ions appear as a result of electron dissociative attachment to oxygen molecules. They are trapped in the ambipolar electric field of the discharge and concentrate themselves near the discharge tube axis. To calculate negative ion density and their spatial distribution, a simple drift-diffusion model of an axially uniform positive column is used.

The model is based on Shottke’s approach and assumes spatially independent kinetic coefficients, zero boundary conditions for positive and negative ion densities on the wall of the discharge tube, Boltzmann distribution of electrons in the radial electric field, and zero negative ion flux on the tube wall. The model is not self-consistent and requires empirical values of an effective electron temperature $T_{e}$, electron density on the tube axis $n_{0}$, and axial electric field $E_{z}$. Only one type of negative ion $O^{-}$ and one type of positive oxygen ion $O_{2}^{+}$ are considered. Atomic negative ions $O^{-}$ prevail over molecular ions $O_{2}^{-}$ in oxygen under the experimental conditions because of the low pressure. Positive ions are represented by $O_{2}^{+}$, since $O_{2}$ has lower ionization energy than the argon or atomic oxygen.

The positive ions are generated in the ionization process with the ionization rate $\nu_{i}$, which is an adjustable parameter (eigenvalue of an equation system). The negative ions appear in the dissociative attachment reactions with ground state and metastable oxygen molecules

(1) $$\begin{array}{c} O_{2}+e\;\overset{k_{a}}{⟶}\;O+O^{-},\;\;O_{2}\left(a^{1}{▵}_{g}\right)+e\;\overset{\;\;k_{\mathrm{am}}}{⟶}\;O+O^{-}. \end{array}$$

Because of low electron density, only the $O_{2}\left(a^{1}{▵}_{g}\right)$ metastable state is considered. This state has extremely small wall quenching efficiency for glass surfaces [6], so the density of $O_{2}\left(a^{1}{▵}_{g}\right)$ is determined by the bulk reactions, while other reactive and excited components quench on the wall effectively. Most important of them are the electron impact excitation

(2) $$O_{2}\left(X^{3}\sum_{g}^{-}\right)+e\;\overset{k_{1}}{⟶}\;O_{2}\left(a^{1}{▵}_{g}\right)+e,$$

and the losses

(3) $$\begin{array}{cc} & O_{2}\left(a^{1}{▵}_{g}\right)+e\;\overset{k_{2}}{⟶}\;O_{2}\left(X^{3}\sum_{g}^{-}\right)+e, \end{array}$$

(4) $$\begin{array}{cc} & O_{2}\left(a^{1}{▵}_{g}\right)+e\;\overset{k_{3}}{⟶}\;2O+e, \end{array}$$

(5) $$\begin{array}{cc} & O_{2}\left(a^{1}{▵}_{g}\right)+e\;\overset{k_{4}}{⟶}\;O+O\left(D\right)+e. \end{array}$$

A balance of the singlet oxygen production and its losses gives their equilibrium fraction

(6) $$\frac{\left[O_{2}\left(a^{1}{▵}_{g}\right)\right]}{\left[O_{2}\right]}=\frac{k_{1}}{k_{am}+k_{2}+k_{3}+k_{4}},$$

which is equal to 13%. The rate coefficients $k_{a}$, $k_{am}$, $k_{1}$, $k_{3}$, and $k_{4}$ are calculated according to Hsu et al. [7], assuming the electron temperature of 4eV. The constant $k_{2}$ is calculated from the detailed balance as $k_{2}=3/2\;k_{1}\exp\left(0.98/T_{e}\right)$.

Negative ions are lost in the processes of recombination with positive ions $O_{2}^{+}$ with a rate $K_{r}=1.5\cdot 10^{-13}\;m^{3}s^{-1}$ [7] and in collisions with $O_{2}\left(a^{1}{▵}_{g}\right)$. The detachment rate constant is taken as $k_{d}=1.9\cdot 10^{-16}\;m^{3}s^{-1}$ according to Belostotsky et al. [8].

The conservation laws for the ion fluxes in the discharge without microparticles are:

(7) $$\begin{array}{cc} & div{\vec{J}}_{+}=\nu_{i}n_{e}-K_{r}n_{i+}n_{i-}\\ & div{\vec{J}}_{-}=\nu_{a}n_{e}-\nu_{d}n_{i-}-K_{r}n_{i+}n_{i-}, \end{array}$$

where $n_{i+}$ is the positive ion density, $n_{i-}$ is the negative ion density, $n_{e}$ is the electron number density, ${\vec{J}}_{\pm }=-D_{\pm }\vec{grad}\left(n_{i\pm }\right)\pm b_{\pm }n_{i\pm }\vec{E}$ are the radial flows of the positive and negative ions, $D_{\pm }$ are the diffusion coefficients of the positive and negative ions, $b_{\pm }$ are the mobilities, and *E* is the electric field. The attachment and detachment rate $\nu_{a}$ and $\nu_{d}$, respectively, are calculated as $\nu_{a}=\left(k_{a}\cdot 0.87+k_{am}\cdot 0.13\right)\left[O_{2}\right]$ and $\nu_{d}=k_{d}\cdot 0.13\left[O_{2}\right]\;c^{-1}$.

Assuming the Boltzmann’s distribution of electrons in the radial electric field, we can write

(8) $$E_{r}=-\frac{T_{e}}{n_{e}}\frac{\partial n_{e}}{\partial r},$$

For the axially symmetric uniform discharge, the Equation (7) can be rewritten as

(9) $$\begin{array}{cc} & \frac{\partial }{r\partial r}\left(r\frac{\partial n_{i+}}{\partial r}+\tau r\frac{n_{i+}}{n_{e}}\frac{\partial n_{e}}{\partial r}\right)=-\frac{\nu_{i}}{D_{+}}n_{e}+\frac{K_{r}}{D_{+}}n_{i-}n_{i+},\\ & \frac{\partial }{r\partial r}\left(r\frac{\partial n_{i-}}{\partial r}-\tau r\frac{n_{i-}}{n_{e}}\frac{\partial n_{e}}{\partial r}\right)=-\frac{\nu_{a}}{D_{-}}n_{e}+\frac{\nu_{d}}{D_{-}}n_{i-}+\frac{K_{r}}{D_{-}}n_{i-}n_{i+}, \end{array}$$

where $\tau =T_{e}/T_{i}$, $T_{i}=0.025$ eV is the ion temperature, and Einstein’s relation for mobilities is used. The Equation (9) should be supplemented by the quasi-neutrality condition $n_{i+}=n_{i-}+n_{e}$.

A special set of variables is convenient for practical calculations:

(10) $$\begin{array}{ccc} & Y_{0} & =n_{e},\\ & Y_{1} & =n_{i-},\\ & Y_{2} & =r\frac{\partial n_{i-}}{\partial r}-\tau r\frac{n_{i-}}{n_{e}}\frac{\partial n_{e}}{\partial r},\\ & Y_{3} & =\left(1+\tau +2\tau \frac{n_{i-}}{n_{e}}\right)r\frac{\partial n_{e}}{\partial r}. \end{array}$$

The variables $Y_{2}$ and $Y_{3}$ are the weighted negative ion flow and difference of the positive and negative ion flows. The system of Equation (9) can be rewritten in terms of the variables (10) and transformed to

(11) $$\begin{array}{ccc} & \frac{\partial Y_{0}}{\partial r} & =\frac{Y_{3}}{r(1+\tau +2\tau Y_{1}/Y_{0})},\\ & \frac{\partial Y_{1}}{\partial r} & =\frac{1}{r}\left(Y_{2}+\frac{\tau Y_{1}Y_{3}}{\left(1+\tau \right)Y_{0}+2\tau Y_{1}}\right),\\ & \frac{\partial Y_{2}}{\partial r} & =r\left(-\frac{\nu_{a}}{D_{-}}Y_{0}+\frac{\nu_{d}}{D_{-}}Y_{1}+\frac{K_{r}n_{0}}{D_{-}}\left(Y_{0}+Y_{1}\right)Y_{1}\right),\\ & \frac{\partial Y_{3}}{\partial r} & =r\left(\left(\frac{\nu_{a}}{D_{-}}-\frac{\nu_{i}}{D_{+}}\right)Y_{0}+\left(\frac{K_{r}}{D_{+}}-\frac{K_{r}}{D_{-}}\right)n_{0}\left(Y_{0}+Y_{1}\right)Y_{1}-\frac{\nu_{d}}{D_{-}}Y_{1}\right). \end{array}$$

The boundary conditions at the tube wall are $Y_{0}=0$ and $Y_{2}=0$, which corresponds to zero electron number density and zero negative ion flux on the tube wall. The conditions on the tube axis are finite values of $Y_{0}$ and $Y_{1}$, and zero for flows $Y_{2}=0$ and $Y_{3}=0$. The electron density at the tube axis can be arbitrary and depends on discharge current. The negative ion density on the axis should be chosen to satisfy the boundary condition for the ion flow at the wall. The boundary condition for the electron density at the wall is ensured by chosen ionization rate $\nu_{i}$.

For estimation of the electron temperature and electron number density, we use the data of the reduced electric field and the mean electron energy for DC discharge in the 30 mm diameter plasma-chemical reactor with argon–oxygen mixtures from [9]. We assume that the electron temperature is 4 eV, and the electron number density on the tube axis changes from $1\cdot 10^{8}\;{\mathrm{cm}}^{-3}$ for the discharge current of 0.2 mA to $5\cdot 10^{8}\;{\mathrm{cm}}^{-3}$ for the discharge current of 1 mA.

## 3. Simulation Results

**Figure 2:**
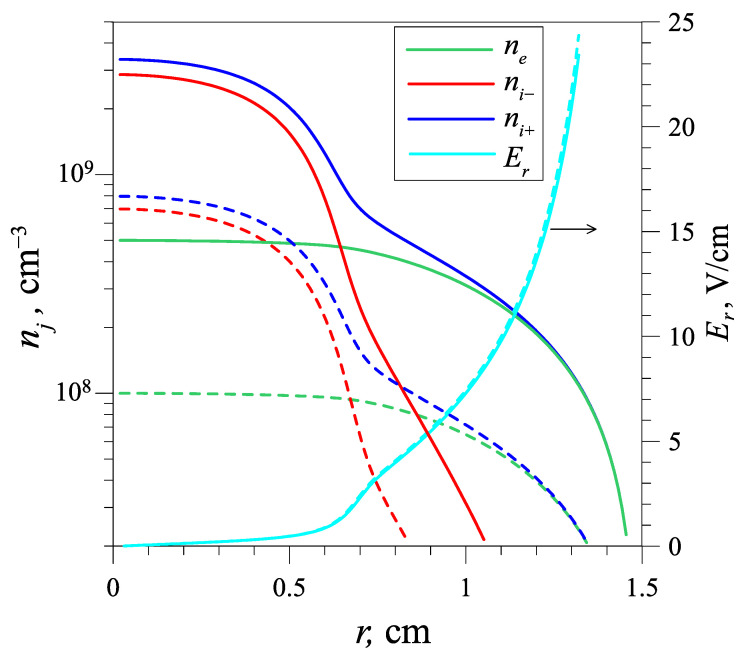
Radial distributions of the electron (green curves), negative ion (red curves), and positive ion (blue curves) densities, as well as the radial electric field (cyan curves) for the DC discharge in the argon diluted by oxygen for the discharge currents of 0.2 (dashed lines) and 1 mA (solid lines).

The calculated radial distributions of positive and negative ion number densities, electron number densities, and the radial electric field for discharge currents 0.2 and 1 mA are presented in Figure 2. The simulation indicates that the narrow negative ion stream forms at the tube axis, and the negative ion density at the tube axis is about seven times higher than the electron density. The radial electric field is almost independent from the discharge current, because recombination of ions gives just a small contribution in ion losses in comparison with detachment process for such low ion densities, so the relative density profiles are almost similar for different currents.

## 4. Discussion

**Figure 3:**
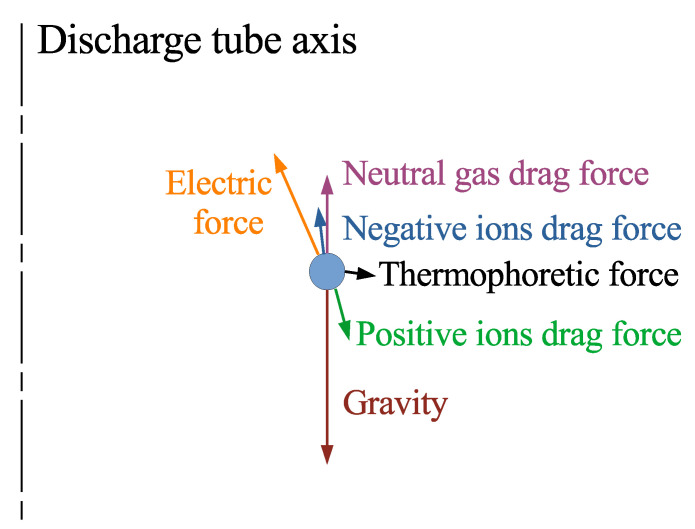
Sketch of principle forces (not to scale) acting on a single microparticle in an oxygen-doped discharge plasma.

The nonuniform spatial distribution of the negative ions is common for different types of discharges. Typically, the discharges in electronegative gases contain electronegative cores and electropositive sheaths [10]. The sheath size is determined by the negative ion production rate and their drift velocity in the ambipolar diffusion field. The electric field in the electropositive sheath is ∼$T_{e}/L_{sh}$, where $L_{sh}$ is the sheath thickness. So, $L_{sh}\approx \sqrt{\tau D_{-}/k_{a}\left[O_{2}\right]}$ and, under the experimental conditions, $L_{sh}∼1\;\mathrm{cm}$, which agrees with the results of our simulation. The microparticle suspension arranged itself in the electropositive sheath of the RF discharge. Conditions of the microparticle levitation require domination of the electric force over the ion drag force. This restricts the possible plasma density in the microparticle structure at the level of $10^{8}$–$10^{9}\;{\mathrm{cm}}^{-3}$ [11]. Thus, the negative ion stream in the DC discharge has a density comparable with the plasma density inside the microparticle cloud and propagates upward into the microparticle suspension. The interaction of this stream with the microparticle suspension can explain the observed influence of the DC discharge on the structure of the suspension [12]. Figure 3 shows schematically the major forces acting on a single microparticle suspended in oxygen-doped discharge plasma. Fluxes of ions, both positive and negative, are directed along the appropriate force and are the primary cause of formation of string-like structures. The negative ions considerably decrease the radial electric field responsible for a microparticle confinement. On the other hand, the ion drift in the axial electric field and the recombination of the positive and negative ions increase gas heating at the axis area, and hence the thermophoretic forces, too, which causes an additional repulsion of the microparticles from the tube axis area.

Aside from decreasing in the microparticle density at the axis area, the negative ion stream also stimulates the formation of string-like structures. The effect of a negative ion flow on an anisotropic potential around a charged absorbing particle has been investigated by Zobnin [13] using a hydrodynamic approach. While the hydrodynamic description is, strictly saying, not valid under the experimental conditions (ion mean free path 0.14mm is comparable to the interparticle distance of about 0.2mm), it nevertheless correctly describes the anisotropic tails of the electric potential around the microparticles on the scale of several interparticle distances. The anisotropic component of the interaction potential decays quite slowly (as 1/r), and therefore can ensure the formation of the string-like structure.

Thus, the proposed model of a gas discharge in the electronegative plasma and the analysis of experimental data show that the intense local flow of negative ions streams in the axial region of DC discharge, which pushes microparticles out of the axial region. For this reason, the void is formed at the axial region of the microparticle suspension already at low discharge current, while in the gas discharge in pure noble gases under the same conditions, there is no void. The presence of electronegative ions also results in an intense string formation. 

## Data Availability

The data are available from the corresponding authors upon reasonable request.

## Sample Availability

Samples of the compounds are not available from the authors.
